# Grains Contribute Shortfall Nutrients and Nutrient Density to Older US Adults: Data from the National Health and Nutrition Examination Survey, 2011–2014

**DOI:** 10.3390/nu10050534

**Published:** 2018-04-25

**Authors:** Yanni Papanikolaou, Victor L. Fulgoni

**Affiliations:** 1 Nutritional Strategies, 59 Marriott Place, Paris, ON N3L 0A3, Canada; 2 Nutrition Impact, 9725 D Drive North, Battle Creek, MI 49014, USA; vic3rd@aol.com

**Keywords:** NHANES, nutrients, aging, grains, fiber

## Abstract

Previous data demonstrate grain foods contribute shortfall nutrients to the diet of U.S. adults. The 2015–2020 Dietary Guidelines for Americans have identified several shortfall nutrients in the U.S. population, including fiber, folate, and iron (women only). Intake of some shortfall nutrients can be even lower in older adults. The present analyses determined the contribution of grain foods for energy and nutrients in older U.S. adults and ranked to all other food sources in the American diet. Analyses of grain food sources were conducted using a 24-hour recall in adults (≥51 years old; *n* = 4522) using data from the National Health and Nutrition Examination Survey, 2011–2014. All grains provided 278 kcal/day or 14% of all energy in the total diet, ranking as the 4th largest contributor of energy compared to 15 main food groups. All grain foods ranked 1st for thiamin (33%) and niacin (23%) intake relative to 15 main food groups. The grain foods category ranked 2nd highest of 15 main food groups for daily dietary fiber (23%), iron (38%), folate (40%), and magnesium (15%) and was the 3rd largest food group contributor for daily calcium intake (13%). When considering nutrients to limit as outlined by dietary guidance, main group of grains contributed 6% total fat, 5% saturated fat, 14% sodium and 9% added sugar. Breads, rolls and tortillas provided 150 kcal/day or 8% of all energy in the total diet, ranking as the 2nd largest contributor of energy compared to 46 food subcategories. Breads, rolls and tortillas ranked 1st of 46 foods for daily thiamin (16%) and niacin (10%) intake and 2nd for dietary fiber (12%), iron (12%), folate (13%), and magnesium (7%). Breads, rolls and tortillas ranked 3rd largest food group contributor for daily calcium (5%) intake. Ready-to-eat cereals provided 47 kcal/day or 2% of all energy in the total diet, ranking as the 20th largest contributor of energy compared to 46 food subcategories. All ready-to-eat cereals ranked 1st for daily iron (19%), 1st for folate (21%), 5th for dietary fiber (7%), 3rd for niacin (9%), 8th for magnesium (4%), and 13th for calcium (2%) intake. Given all grain foods and specific subcategories of grain foods provided a greater percentage of several underconsumed nutrients than calories (including dietary fiber, iron, and folate), grain foods provide nutrient density in the American diet of the older adult.

## 1. Introduction

Food sources of energy and nutrients research in the United States (U.S.) has shown that the grain category can play an important contributory role in the diet of all children, adolescents and adults [1,2]. Specifically, grain foods, relative to energy (kcal) provided a greater percentage of 2015–2020 Dietary Guidelines for Americans (2015–2020 DGA) under-consumed nutrients and nutrients of public health concern [3], including dietary fiber, folate, magnesium, calcium and iron. Breads, rolls, tortillas and ready-to-eat cereals, as subcategories of grain foods, are also meaningful contributors (i.e., ≥10% in the diet) of dietary fiber, thiamin, folate, iron, zinc and niacin to the American diet [1,2]. Further, earlier data from the National Health and Nutrition Examination Survey (NHANES) 2003–2006 in Americans aged ≥2 years old, identified that significant amounts of vitamins A, B6, B12, C and D, as well as thiamin, riboflavin, niacin, folate and iron were from enriched and/or fortified foods with researchers concluding that without enrichment and/or fortification, nutrient shortfalls are further exacerbated [4]. Nevertheless, previous studies have focused on children and all adults (≥19 years old) with no current data isolating older Americans (i.e., >50 years old). Indeed, this demographic represents a significant segment of the U.S. population with the Centers for Disease Control and Prevention (CDC) stating “the growth in the number and proportion of older adults is unprecedented in the history of the U.S. with two factors contributing to the increased number of older adults—increased life spans and aging baby boomers”. The CDC has estimated that the American population for those aged 65 years or older will double in 25 years to approximately 72 million [5]. The 2015–2020 Dietary Guidelines for Americans (2015–2020 DGA) recommends all individuals two years of age and older meet nutrient requirements through foods as part of a nutrient-dense diet; however, many individuals fall short in several nutrients, resulting in the declaration that several nutrients are under-consumed relative to the estimated average requirement (EAR; the average daily nutrient intake level estimated to meet the requirements of half of the healthy individuals in a group) or adequate intake levels established by the National Academy of Medicine (formerly known as the Institute of Medicine) [3].

Indeed, healthy aging is multifactorial, however, nutrition is one of the key determinants of successful aging, as it has been documented to promote health and functionality [6]. Thus, adequate intake of macronutrients and micronutrients represents a key factor in healthy aging. The 2015–2020 DGA identified under-consumed nutrients as shortfall nutrients and included vitamins A, D, E, C, folate, calcium, magnesium, fiber, potassium and iron in specific populations [3]. Similar to the preceding policy and scientific advisory committee reports [7,8], the 2015–2020 DGA continues to highlight calcium, potassium, vitamin D and fiber as shortfall ‘nutrients of public health concern’ in Americans, since under-consumption of these nutrients has been associated with adverse health outcomes. Dietary recommendations further advocate for increased whole grain consumption (i.e., make half your grains whole grains) while limiting enriched/refined grains in the diet [3]. While certain grain food products contribute nutrients to limit in the diet, including added sugar, sodium, and saturated fat, grain foods as a category, including enriched/refined grains also provide a range of beneficial nutrients in the older population, including dietary fiber, calcium, magnesium and B vitamins (thiamin, riboflavin, niacin, cobalamin (B12) and folate) [1,2,9,10]. Similar to all of Americans, fiber is a shortfall nutrient and nutrient of public health concern in older adults, where only 4% of men and 13% of women had a dietary fiber intake above the recommended Adequate Intake (AI) level [11]. The Dietary Guidelines Scientific Advisory Committee has highlighted that calcium intake from foods and beverages did not meet the EAR for older adults, where 71% of men and 81% of females aged ≥71 years old had intakes below the EAR. Similarly, dietary folate and magnesium are underconsumed relative to the Estimated Average Requirement (EAR) in the American population [11]. An analysis using data from the National Health and Nutrition Examination Survey (NHANES) 2009–2012 found that percent of adults below the Estimated Average Requirement (EAR) increased with age for calcium, folate, magnesium, vitamin E and vitamin D [12].

As data in all children and adults support grain food contributions to nutrient density in U.S. dietary patterns and since several nutrients in grains play a distinct role in healthy aging, the objective of the present analyses was to determine sources of energy and nutrients in all grains, and in commonly consumed grain foods (i.e., breads, ready-to-eat cereals, etc.) and rank relative to all other foods in U.S. older adults (≥51 years old) using data from NHANES, 2011–2014. 

## 2. Experimental Section

The NHANES is a nationally-representative, cross-sectional survey of U.S. non-institutionalized, civilian residents. NHANES data are collected by the National Center for Health Statistics of the Centers for Disease Control and Prevention. Written informed consent was obtained for all participants or proxies, and the survey protocol was approved by the Research Ethics Review Board at the National Center for Health Statistics. Data from two NHANES datasets (2011–2012; 2013–2014) were combined for the present analyses in adults ≥51 years old [13]. Nutrient intake data for NHANES 2011–2014 are from the United States Department of Agriculture (USDA) Food and Nutrient Database for Dietary Studies (FNDDS) 5.0 and 6.0 [14]. FNDDS are databases that provide the nutrient values for foods and beverages reported in What We Eat in America (WWEIA), the dietary intake component of NHANES for each data release. The WWEIA Food Categories provide an application to analyze food and beverages as consumed in the American diet. The classification scheme includes 150 unique categories and there are 15 main food groups and 46 subcategories of foods. WWEIA food categories have been previously published by USDA [15].

In the current analyses, each grain food and all other foods reported in WWEIA/NHANES is placed in one of the mutually-exclusive food subcategories by linking each food code contained in FNDDS to one WWEIA category [15]. Mixed grain dishes were separated from the main grains group as a food subcategory, and were included in the current analyses. In the present analyses, the combined NHANES dataset sample included 4522 male and female participants, ≥51 years of age, who had reliable and complete 24-h dietary intake data from WWEIA. Trained individuals complete the 24-h dietary recalls using USDA’s dietary data collection instrument, the Automated Multiple-Pass Method, which includes detailed descriptions of all food and amounts consumed by subjects. While two days of 24-h dietary recalls are collected in WWEIA, the current analysis used Day 1 data as this represents the in-person data collection in the Mobile Examination Center [16].

### Methods and Statistical Analysis

All statistical analyses were performed using SAS software (Version 9.2, SAS Institute, Cary, NC, USA). SAS PROC SURVEYMEANS was used for all statistical calculations, including means and percentages. Survey weights were used to generate nationally-representative estimates for U.S. adults, which were also adjusted for the complex sample design of NHANES. Mean and standard errors of energy, macronutrients and micronutrients for the daily total diet and from food groups were determined (nutrients from dietary supplements were not considered). Percentages of total energy and nutrient intake contributed from food sources were tabulated by ranked order.

## 3. Results

Food sources of energy and nutrients for main food groups are shown in Tables 1–21, which appear in this article. Food sources of energy and nutrients for USDA food subcategories are shown in Supplemental Tables S1–S19 and are available online. All foods are ranked based on energy or nutrient contribution in the total diet. Only values >1.0% contribution to the total diet are reported so as not to consider inconsequential sources.

### 3.1. Main Food Groups and Subcategory Food Groups: Energy Contributions

Energy contribution of main food groups and subcategory of foods groups are presented in Table 1 and Table 2. The greatest main food group contributors of energy in the diet are mixed dishes (19.3%), snacks and sweets (17.0%), protein foods (16.2%), grains (14.1%) and non-alcoholic beverages (7.2%) (Table 1). When considering subcategories of food groups, the top five ranking foods for energy contribution were sweet bakery products (7.8%), breads, rolls, and tortillas (7.6%), plant-based protein foods (4.4%), alcoholic beverages (4.3%) and grain-based mixed dishes (4.2%). Other grain-based foods providing ≤5% of energy in the total diet included, sandwiches (3.1%), ready-to-eat cereals (2.4%), pizza (2.3%), quick breads and bread products (1.6%), cooked grains (1.5%) crackers (1.2%), and cooked cereals (1.1%) (Table 2).

### 3.2. Main Food Groups: 2015–2020 Dietary Guidelines for Americans Shortfall Nutrients

#### 3.2.1. 2015–2020 Dietary Guidelines Nutrients of Public Health Concern: Dietary Fiber, Calcium, Vitamin D, and Potassium

The highest ranking (i.e., ≥10%) main food group contributors, providing 94.31% of dietary fiber in the diet, were grains (23.1%), mixed dishes (19.0%), vegetables (18.3%), fruit (11.7%), snacks and sweets (11.7%), and protein foods (10.6%) (Table 3). The top ranking main food group contributors for calcium, providing 62.9% of calcium in the diet, were milk and dairy (32.8%), mixed dishes (16.9%), and grains (13.2%) (Table 4). When considering vitamin D, the highest ranking main food group contributors providing 71.0% of vitamin D in the diet were milk and dairy (40%) and protein foods (31.0%) (Table 5). The largest main food group contributors of potassium were protein foods (16.8%), mixed dishes (16.3%), non-alcoholic beverages (16.2%), and vegetables (14.7%); collectively, these main food groups contributed 64.0% of potassium in the diet (Table 6).

#### 3.2.2. 2015–2020 Dietary Guidelines Shortfall Nutrients: Folate, Iron, Magnesium, Vitamin A, Vitamin E, and Vitamin C

The largest ranking (i.e., ≥10%) main food group contributors of dietary folate providing 60.3% of folate in the diet, were grains (40.5%), and mixed dishes (19.9%) (Table 7). The top ranking main food group contributors for iron, providing 83.4% of iron in the diet, were grains (37.8%), mixed dishes (19.6%), protein foods (14.4%), and snacks and sweets (11.7%) (Table 8). For magnesium, the highest ranking main food group contributors providing 59.5% of magnesium in the diet were protein foods (17.7%), grains (15.0%), mixed dishes (14.6%) and non-alcoholic beverages (12.2%) (Table 9). The largest main food group contributors of vitamin A were vegetables (23.0%), milk and dairy (15.9%), mixed dishes (14.2%), grains (12.9%), and protein foods (11.6%), collectively, providing 77.7% of vitamin A in the diet (Table 10). The greatest main food group sources of vitamin E, contributing 78.3% of vitamin E in the diet, were protein foods (22.9%), mixed dishes (17.4%), snacks and sweets (16.1%), vegetables (11.4%), and grains (10.5%) (Table 11). The top three main food group sources of vitamin C were non-alcoholic beverages (36.0%), vegetables (25.6%), and fruit (18.2%), contributing a total of 79.8% of all vitamin C in the total daily diet (Table 12).

### 3.3. Subcategory Food Groups: 2015–2020 Dietary Guidelines for Americans’ Shortfall Nutrients

#### 3.3.1. 2015–2020 Dietary Guidelines Nutrients of Public Health Concern: Dietary Fiber, Calcium, Vitamin D, and Potassium

The top ranking (i.e., ≥10%) subcategory foods, collectively providing 37.4% of dietary fiber to the total diet, were vegetables, excluding potatoes (13.6%), breads, rolls and tortillas (12.0%) and fruits (11.7%) (Table S1). When considering calcium, two food groups contributed approximately a quarter of all calcium in the diet (27.5%) milk (15.7%) and cheese (11.8%) (Table S2). When considering vitamin D (Table S3), the highest ranking subcategory contributors providing 47.4% of vitamin D in the diet were milk (30.1%) and seafood (17.4%). While no one food item provided ≥10% of potassium in the total diet, coffee, vegetables (excluding potatoes), fruits, milk, white potatoes, meat/poultry/fish mixed dishes, plant-based protein foods, 100% juice, breads, rolls, and tortillas, and poultry were the top ten ranking contributors, collectively providing 57.3% of all potassium in the diet (Table S4). 

#### 3.3.2. 2015–2020 Dietary Guidelines Shortfall Nutrients: Folate, Iron, Magnesium, Vitamin A, Vitamin E, and Vitamin C

Two grain food groups were the two largest contributors (i.e., ≥10%) of dietary folate as Dietary Folate Equivalents (DFE) providing 34.4% of folate in the diet, namely ready-to-eat cereals (21.0%), and breads, rolls and tortillas (13.4%) (Table S5). Similarly, ready-to-eat cereals, and breads, rolls, and tortillas provided nearly one-third of all iron in the total diet (30.8%) (Table S6). While no one food item provided ≥10% of all magnesium in the total diet, plant-based protein foods, breads, rolls, and tortillas, coffee and tea, vegetables (excluding potatoes), plain water, milk, fruits, ready-to-eat cereals, meat/poultry/fish mixed dishes, and grain-based mixed dishes were the top ten ranking contributors, collectively providing 53.6% of all magnesium in the diet (Table S7). Only vegetables, excluding potatoes, provided ≥10% of vitamin A in the total diet (21.7%). An additional 47.2% of all vitamin A in the diet was providing by the following nine foods: milk, ready-to-eat cereals, fats and oils, eggs, meats, cheese, desserts, meat/poultry/fish mixed dishes, and grain-based mixed dishes (Table S8). The top ten ranking foods for vitamin E contributed 61.9% (Table S9) of vitamin E in the diet, however, the category of plant-based protein foods was the only food source to provide ≥10% of all vitamin E (12.7%). The top three ranking foods for vitamin C (Table S10) provided 65.4% of all vitamin C in the diet: 100% juice (25.4%), vegetables, excluding potatoes (21.8%), and fruits (18.2%).

### 3.4. Main Food Groups: Total Fat, Saturated Fat, Added Sugars and Sodium Contributions

Protein foods, mixed dishes, snacks and sweets were the source for nearly 77% of total fat in the diet (Table 13). Mixed dishes, snacks and sweets, protein foods, milk and dairy, and fats and oils contributed 88% of all saturated fat in the total diet (Table 14). Two main food groups, non-alcoholic beverages, and snacks and sweets contributed nearly 66% of added sugars in the diet (Table 15). The three largest main food group sources of sodium in the total diet were mixed dishes (29.2%), protein foods (22.7%), and grains (14.1%) (Table 16).

### 3.5. Subcategory Food Groups: Total Fat, Saturated Fat, Added Sugars and Sodium Contributions

While no one subcategory of food contributed ≥10% of all total fat (Table S11) in the diet, the top ten food sources collectively provided 56.9% of total fat, with fats and oils and sweet bakery products being the largest contributors. Likewise, while fats and oils, and sweet bakery products were the largest contributors of saturated fat (9.5% and 9.2%, respectively), no food item contributed ≥10% of all saturated fat in the diet (Table S12). Two food groups provided nearly half of all added sugars in the total diet (Table S13)—sweetened beverages (25.0%) and sweet bakery products (17.2%). Similar to total fat and saturated fat, there was no food group that provided ≥10% of all sodium in the diet (Table S14). The top ten foods, collectively contributing 53.6% of all sodium in the total diet, were breads, rolls, and tortillas, cured meats/poultry, meat/poultry/fish mixed dishes, soups, grain-based mixed dishes, vegetables (excluding potatoes), poultry, condiments and sauces, sandwiches, and sweet bakery products.

### 3.6. Main Food Groups: Thiamin, Niacin, Riboflavin, Vitamin B6, Vitamin B12

Four main food groups contribute nearly 78% of all thiamin in the diet, with the leading main food group being grain foods, providing 32.6% of thiamin in the total diet (Table 17). For niacin, the top three contributors in the diet were protein foods (29.1%), mixed dishes (22.6%) and grains (22.5%), collectively providing nearly 75% of niacin in the diet (Table 18). The top five contributors of riboflavin, collectively providing 81.2% of riboflavin were non-alcoholic beverages, grains, milk and dairy, protein foods, and mixed dishes (Table 19). Protein foods, grains, mixed dishes and vegetables were the largest contributors of vitamin B6 (72.7%) (Table 20), while protein foods, mixed dishes, milk and dairy, and grains contributed nearly 90% of all vitamin B12 in the diet (Table 21).

### 3.7. Subcategory Food Groups: Thiamin, Niacin, Riboflavin, Vitamin B6, Vitamin B12

Two grain subcategories (i.e., breads, rolls and tortillas, and ready-to-eat cereals) were the largest contributors (≥10% for each food) of thiamin in the diet, collectively providing more than a quarter of thiamin in the total diet (Table S15). Breads, rolls, and tortillas were the only food to provide ≥10% of niacin in the total diet (Table S16). The top two contributors of riboflavin in the total diet were coffee and tea, and milk, collectively providing 23.8% of riboflavin in the diet (Table S17). Ready-to-eat cereals were the sole food subcategory to provide ≥10% of vitamin B6 in the total diet (Table S18), while meats, ready-to-eat cereals, and milk were the top-ranking subcategory of foods for vitamin B12, collectively providing 42.0% of all vitamin B12 in the total diet (Table S19).

## 4. Discussion

Americans of all ages are continuously not meeting nutrition recommendations even as previous [8] and current dietary guidelines [3] have identified shortfall and nutrients of public health concern. The current analyses focused on older American adults, as sources of energy and nutrients in this population remain limited. The present analyses determined the contribution of grain foods to energy and nutrients in older U.S. adults and ranked other food sources in the American diet, to provide an assessment of energy and nutrient contribution to the total diet. While all grain foods contributed 14% of all energy in the total diet, ranking as the 4th largest contributor of energy compared to 15 main food groups, grain foods can provide nutrient density to the American diet of older adults given they provide a greater percentage of nutrients than energy to the diet. The grain foods category ranked 2nd highest of 15 main food groups for daily dietary fiber, iron, folate, and magnesium, the 3rd largest main food group contributor for daily calcium intake (13 ± 0.4%) and 1st for thiamin and niacin intake. When evaluating subcategories of foods, breads, rolls and tortillas provided 150 ± 2.8 kcal/day or 8% of all energy in the total diet, ranking as the 2nd largest contributor of energy compared to 46 food subcategories. The grain subcategory of breads, rolls and tortillas ranked 1st of 46 food subcategories for daily thiamin and niacin intake and 2nd for dietary fiber, iron, folate, and magnesium. Breads, rolls and tortillas ranked 3rd largest food subcategory contributor for daily calcium intake. Ready-to-eat cereals provided 47 ± 2.6 kcal/day or 2% of all energy in the total diet, ranking as the 20th largest contributor of energy compared to 46 food subcategories. All ready-to-eat cereals ranked 1st for daily iron, 1st for folate, 5th for dietary fiber, 3rd for niacin, 8th for magnesium, and 13th for calcium intake. When considering nutrients to limit as outlined by dietary guidance, main group of grains contributed 6.27% total fat, 4.58% saturated fat, 14.13% sodium and 8.9% added sugar.

Current and preceding dietary guidance [3,8] for Americans recommend that half of all grains consumed be whole grains, with limitations placed on refined/enriched grain consumption. This may be promoting a perception that non-whole grain foods are not a component of a healthy dietary pattern, due to minimal or less nutrient density and increased caloric intake. The current analysis considered all foods consumed in the American diet, including all grain foods. Additionally, the current analysis did not separate whole- and refined-grain foods, as a key objective included determining the sources of energy and nutrients from all foods, and grain foods in particular, as currently consumed in the older American population. As such, the analysis showed that any perceptions that refined grain foods may be lacking in nutrient density are invalid. Indeed, certain grain foods (i.e., sweet bakery products) in the total diet can contribute nutrients to limit, including added sugar, sodium, and saturated fat and hence, these grain foods should be consumed in moderation and within caloric recommendations as designated by dietary guidance to promote public health. Nonetheless, the entire grain food category is the first, second, or third ranking category delivering dietary fiber, folate, iron, magnesium, calcium, and vitamin D—all nutrients identified as shortfall nutrients by the 2015–2020 DGA [3]. Similarly, further analysis with specific food subcategories within the grain category show that breads, rolls and tortillas, grain foods routinely consumed in the U.S. diet, are also first, second and/or third ranking foods for dietary fiber, folate, calcium, iron, magnesium, vitamin A, for less than 8.5% of all calories and sodium, less than 3% of total fat, less than 2.5% of all saturated fat, and less than 3.5% of all added sugar in the total diet. Comparably, the current data provide sources of energy and nutrients data in the older American adult for ready-to-eat cereals, another commonly consumed grain food in the U.S. Ready-to-eat cereals are first, second, third, fourth, and/or fifth ranking foods for dietary fiber, folate, iron, vitamin A, vitamin D for less than 2.5% of all calories and sodium, less than 1% of total fat, less than 0.5% of all saturated fat, and less than 4% of all added sugar in the total diet.

Many older Americans are not meeting recommendations for several key nutrients, many of which are contributed by grain food products. Thus, maintaining both whole- and refined/enriched grains, as part of a healthy dietary pattern, may prove to benefit nutrient intakes in a substantial portion of the U.S. older population. The 2015–2020 DGAs identified shortfall nutrients in all Americans 2 years of age, however, intake of shortfall nutrients can be even lower in older adults. A recent NHANES analysis examining food and supplement use in various age groups found significant differences in the proportion of the population with intakes below EAR. Adults ≥71 years of age had a higher prevalence of inadequacy for calcium, magnesium, and most B vitamins compared to middle-aged and younger adults. Overall, when considering food consumption alone, the data showed that older adults had lower intake and higher prevalence of inadequacy for most nutrients relative to younger adults, with use of dietary supplements being associated with the greatest benefit in older adults [12]. An Irish cross-sectional analysis that explored dietary intakes of community-dwelling elderly individuals found that a substantial number of participants had inadequate intakes of several nutrients, including calcium, magnesium, vitamin D, folate, zinc and vitamin C [17]. U.S. national surveys of dietary intake consistently report all Americans, including older adults, do not meet recommendations for dietary fiber intake [18]. A review by Allen (2009) reported that in the U.S. and the United Kingdom, approximately 6% of adults ≥60 years of age are vitamin B12 deficient, with prevalence of deficiency increasing as individuals age [19]. Another cross-sectional study of free-living New York city older adults participating in the Cardiovascular Health of Seniors and Built Environment Study, found that both genders fell short on recommended intake for fiber, calcium, magnesium, potassium, zinc, folate, and vitamins A, B6, C, D, and E, and in some instances, women had intakes for thiamin below the recommendations [20]. Similarly, a recent systematic review identified inadequate intake of vitamin D, thiamin, riboflavin, calcium, magnesium, and selenium in community-dwelling older adults from North America, Europe, Australia and New Zealand [21] population. 

The current analyses have a limitation inherent in observational research and has previously been reported in similar research designs. The results are dependent on self-reported dietary data for foods, which may involve study participants under- or over-estimating food consumption, leading to inaccuracies in energy and nutrient intakes. Data were also obtained using a 24-hour dietary recall, which relies on study participant memory, and while validated methods are used to gather the data, recall information is subject to inaccuracies and bias from memory challenges and other potential measurement errors experienced in epidemiological investigations [22]. Caution should be administered when comparing the current findings to previous studies, as food groupings can impact sources of energy and nutrient outcomes, specifically where there are differences in the level of aggregation (i.e., the number of food groups) or disaggregation methods used by researchers. A significant benefit of using NHANES data for the current analyses includes access to a large and nationally-representative dataset of adults of various age groups in the U.S. and corresponding food and nutrient intake data.

## 5. Conclusions

Although existing dietary guidance recommends an increase in shortfall nutrients and nutrients of public health concern, predominantly through food consumption, gaps in nutrient intakes persist in older U.S. adults. The current sources of energy and nutrients analyses show that cumulatively, a variety of grain foods, regularly consumed by older American adults, in addition to other nutrient-dense foods, including fruits and vegetables, help contribute to nutrient density in the total diet. Specifically, grain foods are contributors (≥10% in the total diet) of the 2015–2020 DGA under-consumed shortfall nutrients and nutrients of public health concern, including dietary fiber, folate, magnesium, calcium and iron, vitamin A, vitamin E, vitamin B12, niacin, and thiamin. The subcategory of breads, rolls and tortillas are contributors of daily thiamin, niacin, dietary fiber, folate, and iron, while ready-to-eat cereals contribute iron, folate, thiamin, vitamin B6, and vitamin B12. Based on the current data presented, encouraging certain grain food patterns in older U.S. adults, including selecting a mix of whole-, enriched- and fortified-grains, from breads, and cereals, may improve nutrient intakes and minimize gaps in shortfall nutrient intakes. In contrast, eliminating grains, especially refined grains, from the diet may lead to unintended nutrient intake consequences. Developing dietary strategies that are mindful of recommended caloric needs, while monitoring nutrients to limit (i.e., added sugars, sodium and saturated fat) can benefit from the inclusion of refined/enriched and whole grain selections in the U.S. diet.

## Figures and Tables

**Table 1:** Main food group sources of energy (kcal) contribution in the total U.S. diet, adults ≥51 years old (*n =* 4522; gender combined; daily intake data; National Health and Nutrition Examination Survey. (NHANES) 2011–2014; main food groups contributing <1.0% not reported; data are Day 1 intakes).

Main Food Group	Rank	% Energy Contribution
Mixed Dishes	1	19.34
Snacks and Sweets	2	16.97
Protein Foods	3	16.15
Grains	4	14.06
Beverages, Nonalcoholic	5	7.16
Milk and Dairy	6	6.47
Vegetables	7	5.94
Alcoholic Beverages	8	4.34
Fats and Oils	9	3.88
Fruit	10	3.17
Sugars	11	1.37

**Table 2:** Subcategory food group sources of energy (kcal) contribution in the total U.S. diet, adults ≥51 years old (*n =* 4522; gender combined; daily intake data; NHANES 2011–2014; food subcategories contributing <1.0% not reported; data are Day 1 intakes).

Food Group	Rank	% Energy Contribution
Sweet Bakery Products	1	7.77
Breads, Rolls, Tortillas	2	7.58
Plant-based Protein Foods	3	4.40
Alcoholic Beverages	4	4.34
Mixed Dishes—Grain-based	5	4.22
Mixed Dishes—Meat, Poultry, Fish	6	4.12
Sweetened Beverages	7	4.00
Fats and Oils	8	3.88
Fruits	9	3.17
White Potatoes	10	3.16
Mixed Dishes - Sandwiches	11	3.13
Poultry	12	3.04
Savory Snacks	13	3.03
Milk	14	2.81
Vegetables, excluding Potatoes	15	2.77
Mixed Dishes—Mexican	16	2.74
Other Desserts	17	2.66
Meats	18	2.60
Cured Meats/Poultry	19	2.47
Ready-to-Eat Cereals	20	2.35
Mixed Dishes—Pizza	21	2.26
Cheese	22	2.22
Eggs	23	2.07
Candy	24	2.02
Quick Breads and Bread Products	25	1.60
Seafood	26	1.57
Mixed Dishes—Soups	27	1.56
Coffee and Tea	28	1.54
100% Juice	29	1.52
Cooked Grains	30	1.46
Sugars	31	1.37
Mixed Dishes—Asian	32	1.31
Crackers	33	1.15
Cooked Cereals	34	1.07

**Table 3:** Food sources of dietary fiber contribution in the total U.S. diet, adults ≥51 years old (*n =* 4522; gender combined; daily intake data; NHANES 2011–2014; main food groups contributing <1.0% not reported; data are Day 1 intakes).

Main Food Group	Rank	% Fiber Contribution
Grains	1	23.12
Mixed Dishes	2	18.95
Vegetables	3	18.25
Fruit	4	11.70
Snacks and Sweets	5	11.69
Protein Foods	6	10.61
Beverages, Nonalcoholic	7	2.38
Condiments and Sauces	8	1.97

**Table 4:** Food sources of calcium contribution in the total U.S. diet, adults ≥51 years old (*n =* 4522; gender combined; daily intake data; NHANES 2011–2014; main food groups contributing <1.0% not reported; data are Day 1 intakes).

Main Food Group	Rank	% Calcium Contribution
Milk and Dairy	1	32.77
Mixed Dishes	2	16.86
Grains	3	13.24
Snacks and Sweets	4	8.00
Beverages, Nonalcoholic	5	7.69
Water	6	6.33
Protein Foods	7	5.90
Vegetables	8	4.95
Fruit	9	1.22

**Table 5:** Food sources of vitamin D (D2 + D3) contribution in the total U.S. diet, adults ≥51 years old (*n =* 4522; gender combined; daily intake data; NHANES 2011–2014; DFE = dietary folate equivalents; main food groups contributing <1.0% not reported; data are Day 1 intakes).

Main Food Group	Rank	% Vitamin D Contribution
Milk and Dairy	1	40.06
Protein Foods	2	30.95
Grains	3	9.33
Mixed Dishes	4	8.19
Beverages, Nonalcoholic	5	5.71
Snacks and Sweets	6	2.14
Vegetables	7	1.56
Fats and Oils	8	1.25

**Table 6:** Food sources of potassium contribution in the total U.S. diet, adults ≥51 years old (*n =* 4522; gender combined; daily intake data; NHANES 2011–2014; main food groups contributing <1.0% not reported; data are Day 1 intakes).

Main Food Group	Rank	% Potassium Contribution
Protein Foods	1	16.75
Mixed Dishes	2	16.32
Beverages, Nonalcoholic	3	16.17
Vegetables	4	14.73
Milk and Dairy	5	9.05
Snacks and Sweets	6	8.01
Fruit	7	7.18
Grains	8	6.66
Alcoholic Beverages	9	2.18
Condiments and Sauces	10	1.60

**Table 7:** Food sources of folate, DFE, contribution in the total U.S. diet, adults ≥51 years old (*n =* 4522; gender combined; daily intake data; NHANES 2011–2014; DFE = dietary folate equivalents; main food groups contributing <1.0% not reported; data are Day 1 intakes).

Main Food Group	Rank	% Folate, DFE Contribution
Grains	1	40.48
Mixed Dishes	2	19.85
Snacks and Sweets	3	9.69
Vegetables	4	9.46
Protein Foods	5	7.39
Beverages, Nonalcoholic	6	6.21
Fruit	7	2.22
Milk and Dairy	8	2.06
Alcoholic Beverages	9	1.39

**Table 8:** Food sources of iron contribution in the total U.S. diet, adults ≥51 years old (*n =* 4522; gender combined; daily intake data; NHANES 2011–2014; main food groups contributing <1.0% not reported; data are Day 1 intakes).

Main Food Group	Rank	% Iron Contribution
Grains	1	37.78
Mixed Dishes	2	19.61
Protein Foods	3	14.37
Snacks and Sweets	4	11.67
Vegetables	5	5.98
Beverages, Nonalcoholic	6	4.75
Fruit	7	1.72
Milk and Dairy	8	1.17
Condiments and Sauces	9	1.01

**Table 9:** Food sources of magnesium contribution in the total U.S. diet, adults ≥51 years old (*n =* 4522; gender combined; daily intake data; NHANES 2011–2014; main food groups contributing <1.0% not reported data are Day 1 intakes).

Main Food Group	Rank	% Magnesium Contribution
Protein Foods	1	17.69
Grains	2	15.00
Mixed Dishes	3	14.59
Beverages, Nonalcoholic	4	12.19
Snacks and Sweets	5	9.83
Vegetables	6	9.13
Milk and Dairy	7	7.15
Water	8	4.62
Fruit	9	4.20
Alcoholic Beverages	10	3.23
Condiments and Sauces	11	1.14

**Table 10:** Food sources of vitamin A, retinol activity equivalents (RAE), contribution in the total U.S. diet, adults ≥51 years old (*n =* 4522; gender combined; daily intake data; NHANES 2011–2014; main food groups contributing <1.0% not reported; data are Day 1 intakes).

Main Food Group	Rank	% Vitamin A, RAE Contribution
Vegetables	1	23.01
Milk and Dairy	2	15.94
Mixed Dishes	3	14.19
Grains	4	12.93
Protein Foods	5	11.60
Snacks and Sweets	6	7.19
Beverages, Nonalcoholic	7	6.02
Fats and Oils	8	5.28
Fruit	9	2.16

**Table 11:** Food sources of vitamin E, as alpha-tocopherol, contribution in the total U.S. diet, adults ≥51 years old (*n =* 4522; gender combined; daily intake data; NHANES 2011–2014; main food groups contributing <1.0% not reported; data are Day 1 intakes).

Main Food Group	Rank	% Vitamin E Contribution
Protein Foods	1	22.88
Mixed Dishes	2	17.38
Snacks and Sweets	3	16.11
Vegetables	4	11.44
Grains	5	10.47
Fats and Oils	6	7.37
Beverages, Nonalcoholic	7	5.06
Fruit	8	2.63
Milk and Dairy	9	2.57
Condiments and Sauces	10	2.48

**Table 12:** Food sources of vitamin C contribution in the total U.S. diet, adults ≥51 years old (*n =* 4522; gender combined; daily intake data; NHANES 2011–2014; main food groups contributing <1.0% not reported; data are Day 1 intakes).

Main Food Group	Rank	% Vitamin C Contribution
Beverages, Nonalcoholic	1	35.98
Vegetables	2	25.60
Fruit	3	18.22
Mixed Dishes	4	9.01
Grains	5	3.39
Snacks and Sweets	6	2.50
Condiments and Sauces	7	1.98
Protein Foods	8	1.07

**Table 13:** Food sources of total fat contribution in the total U.S. diet, adults ≥51 years old (*n =* 4522; gender combined; daily intake data; NHANES 2011–2014; main food groups contributing <1.0% not reported; data are Day 1 intakes).

Main Food Group	Rank	% Total Fat Contribution
Protein Foods	1	25.31
Mixed Dishes	2	22.52
Snacks and Sweets	3	19.52
Fats and Oils	4	9.57
Milk and Dairy	5	7.51
Vegetables	6	6.35
Grains	7	6.27
Condiments and Sauces	8	1.41
Beverages, Nonalcoholic	9	1.02

**Table 14:** Food sources of saturated fat contribution in the total U.S. diet, adults ≥51 years old (*n =* 4522; gender combined; daily intake data; NHANES 2011–2014; main food groups contributing <1.0% not reported; data are Day 1 intakes).

Main Food Group	Rank	% Saturated Fat Contribution
Mixed Dishes	1	23.47
Snacks and Sweets	2	21.18
Protein Foods	3	20.19
Milk and Dairy	4	14.08
Fats and Oils	5	9.52
Grains	6	4.58
Vegetables	7	4.44
Condiments and Sauces	8	1.32

**Table 15:** Food sources of added sugars contribution in the total U.S. diet, adults ≥51 years old (*n =* 4522; gender combined; daily intake data; NHANES 2011–2014; main food groups contributing <1.0% not reported; data are Day 1 intakes).

Main Food Group	Rank	% Added Sugar Contribution
Beverages, Nonalcoholic	1	33.25
Snacks and Sweets	2	32.69
Sugars	3	9.09
Grains	4	8.90
Milk and Dairy	5	3.90
Fats and Oils	6	2.78
Mixed Dishes	7	2.71
Protein Foods	8	1.63
Fruit	9	1.43
Alcoholic Beverages	10	1.21

**Table 16:** Food sources of sodium contribution in the total U.S. diet, adults ≥51 years old (*n =* 4522; gender combined; daily intake data; NHANES 2011–2014; main food groups contributing <1.0% not reported; data are Day 1 intakes).

Main Food Group	Rank	% Sodium Contribution
Mixed Dishes	1	29.19
Protein Foods	2	22.68
Grains	3	14.13
Snacks and Sweets	4	8.57
Vegetables	5	8.10
Milk and Dairy	6	5.64
Condiments and Sauces	7	4.31
Fats and Oils	8	3.40
Beverages, Nonalcoholic	9	2.23
Water	10	1.14

**Table 17:** Food sources of thiamin contribution in the total U.S. diet, adults ≥51 years old (*n =* 4522; gender combined; daily intake data; NHANES 2011–2014; main food groups contributing <1.0% not reported; data are Day 1 intakes).

Main Food Group	Rank	% Thiamin Contribution
Grains	1	32.56
Mixed Dishes	2	21.77
Protein Foods	3	13.60
Snacks and Sweets	4	10.06
Beverages, Nonalcoholic	5	6.60
Vegetables	6	6.55
Milk and Dairy	7	4.16
Fruit	8	2.30

**Table 18:** Food sources of niacin contribution in the total U.S. diet, adults ≥51 years old (*n =* 4522; gender combined; daily intake data; NHANES 2011–2014; main food groups contributing <1.0% not reported; data are Day 1 intakes).

Main Food Group	Rank	% Niacin Contribution
Protein Foods	1	29.11
Mixed Dishes	2	22.57
Grains	3	22.52
Snacks and Sweets	4	7.06
Beverages, Nonalcoholic	5	6.77
Vegetables	6	5.25
Alcoholic Beverages	7	2.43
Fruit	8	1.66

**Table 19:** Food sources of riboflavin contribution in the total U.S. diet, adults ≥51 years old (*n =* 4522; gender combined; daily intake data; NHANES 2011–2014; main food groups contributing <1.0% not reported; data are Day 1 intakes).

Main Food Group	Rank	% Riboflavin Contribution
Beverages, Nonalcoholic	1	17.85
Grains	2	17.08
Milk and Dairy	3	15.95
Protein Foods	4	15.64
Mixed Dishes	5	14.70
Snacks and Sweets	6	8.94
Vegetables	7	4.18
Fruit	8	1.95
Alcoholic Beverages	9	1.51

**Table 20:** Food sources of vitamin B6 contribution in the total U.S. diet, adults ≥51 years old (*n =* 4522; gender combined; daily intake data; NHANES 2011–2014; main food groups contributing <1.0% not reported; data are Day 1 intakes).

Main Food Group	Rank	% Vitamin B6 Contribution
Protein Foods	1	24.39
Grains	2	20.11
Mixed Dishes	3	16.51
Vegetables	4	11.70
Beverages, Nonalcoholic	5	5.99
Fruit	6	5.92
Snacks and Sweets	7	4.99
Milk and Dairy	8	3.55
Alcoholic Beverages	9	3.11
Fats and Oils	10	1.45
Condiments and Sauces	11	1.29

**Table 21:** Food sources of vitamin B12 contribution in the total U.S. diet, adults ≥51 years old (*n =* 4522; gender combined; daily intake data; NHANES 2011–2014; main food groups contributing <1.0% not reported; data are Day 1 intakes).

Main Food Group	Rank	% Vitamin B12 Contribution
Protein Foods	1	33.57
Mixed Dishes	2	19.01
Milk and Dairy	3	18.77
Grains	4	17.61
Beverages, Nonalcoholic	5	4.12
Snacks and Sweets	6	3.46
Fats and Oils	7	1.06
Vegetables	8	1.03

## Supplementary Materials

The following are available online at http://www.mdpi.com/2072-6643/10/5/534/s1, Table S1: Subcategory food sources of dietary fiber contribution in the total U.S. diet, adults ≥51 years old (*n* = 4522; gender combined; daily intake data; NHANES 2011–2014; subcategory groups contributing <1.0% not reported; data are Day 1 intakes), Table S2: Subcategory food sources of calcium contribution in the total U.S. diet, adults ≥51 years old (*n* = 4522; gender combined; daily intake data; NHANES 2011–2014; subcategory groups contributing <1.0% not reported; data are Day 1 intakes), Table S3: Subcategory food sources of vitamin D (D2+D3) contribution in the total U.S. diet, adults ≥51 years old (*n* = 4522; gender combined; daily intake data; NHANES 2011–2014; subcategory groups contributing <1.0% not reported; data are Day 1 intakes), Table S4: Subcategory food sources of potassium contribution in the total U.S. diet, adults ≥51 years old (*n* = 4522; gender combined; daily intake data; NHANES 2011–2014; subcategory groups contributing <1.0% not reported; data are Day 1 intakes), Table S5: Subcategory food sources of folate, DFE, contribution in the total U.S. diet, adults ≥51 years old (*n* = 4522; gender combined; daily intake data; NHANES 2011–2014; subcategory groups contributing <1.0% not reported; data are Day 1 intakes), Table S6: Subcategory food sources of iron contribution in the total U.S. diet, adults ≥51 years old (*n* = 4522; gender combined; daily intake data; NHANES 2011–2014; subcategory groups contributing <1.0% not reported; data are Day 1 intakes), Table S7: Subcategory food sources of magnesium contribution in the total U.S. diet, adults ≥51 years old (*n* = 4522; gender combined; daily intake data; NHANES 2011–2014; subcategory groups contributing <1.0% not reported; data are Day 1 intakes), Table S8: Subcategory food sources of vitamin A, RAE, contribution in the total U.S. diet, adults ≥51 years old (*n* = 4522; gender combined; daily intake data; NHANES 2011–2014; subcategory groups contributing <1.0% not reported; data are Day 1 intakes), Table S9: Subcategory food sources of vitamin E, as alpha tocopherol, contribution in the total U.S. diet, adults ≥51 years old (*n* = 4522; gender combined; daily intake data; NHANES 2011–2014; subcategory groups contributing <1.0% not reported; data are Day 1 intakes), Table S10: Subcategory food sources of vitamin C contribution in the total U.S. diet, adults ≥51 years old (*n* = 4522; gender combined; daily intake data; NHANES 2011–2014; subcategory groups contributing <1.0% not reported; data are Day 1 intakes), Table S11: Food sources of total fat contribution in the total U.S. diet, adults ≥51 years old (*n* = 4522; gender combined; daily intake data; NHANES 2011–2014; subcategory groups contributing <1.0% not reported; data are Day 1 intakes), Table S12: Food sources of saturated fat contribution in the total U.S. diet, adults ≥51 years old (*n* = 4522; gender combined; daily intake data; NHANES 2011–2014; subcategory groups contributing <1.0% not reported; data are Day 1 intakes), Table S13: Food sources of added sugar contribution in the total U.S. diet, adults ≥51 years old (*n* = 4522; gender combined; daily intake data; NHANES 2011-2014; subcategory groups contributing <1.0% not reported; data are Day 1 intakes), Table S14: Food sources of sodium contribution in the total U.S. diet, adults ≥51 years old (*n* = 4522; gender combined; daily intake data; NHANES 2011–2014; subcategory groups contributing <1.0% not reported; data are Day 1 intakes), Table S15: Food sources of thiamin contribution in the total U.S. diet, adults ≥51 years old (*n* = 4522; gender combined; daily intake data; NHANES 2011–2014; subcategory groups contributing <1.0% not reported; data are Day 1 intakes), Table S16: Food sources of niacin contribution in the total U.S. diet, adults ≥51 years old (*n* = 4522; gender combined; daily intake data; NHANES 2011–2014; subcategory groups contributing <1.0% not reported; data are Day 1 intakes), Table S17: Food sources of riboflavin contribution in the total U.S. diet, adults ≥51 years old (*n* = 4522; gender combined; daily intake data; NHANES 2011–2014; subcategory groups contributing <1.0% not reported; data are Day 1 intakes), Table S18: Food sources of vitamin B6 contribution in the total U.S. diet, adults ≥51 years old (*n* = 4522; gender combined; daily intake data; NHANES 2011–2014; subcategory groups contributing <1.0% not reported; data are Day 1 intakes), Table S19: Food sources of vitamin B12 contribution in the total U.S. diet, adults ≥51 years old (*n* = 4522; gender combined; daily intake data; NHANES 2011–2014; subcategory groups contributing <1.0% not reported; data are Day 1 intakes).
