# Supplementary material for: Grains Contribute Shortfall Nutrients and Nutrient Density to Older US Adults: Data from the National Health and Nutrition Examination Survey, 2011–2014
Source: Nutrients. 2018 Apr 25;10(5):534. doi: 10.3390/nu10050534 (PMC5986414; doi:10.3390/nu10050534)
Supplement: Supplementary file 1 [file nutrients-10-00534-s001.zip › nutrients-291309-SI.docx]

**Supplemental Tables**

**Table S1.** Subcategory food sources of dietary fiber contribution in the total US diet, adults ≥51 years old (*N =* 4522; gender combined; daily intake data; NHANES 2011-2014; subcategory groups contributing <1.0% not reported; data are Day 1 intakes).

**Table S2.** Subcategory food sources of calcium contribution in the total US diet, adults ≥51 years old (*N =* 4522; gender combined; daily intake data; NHANES 2011-2014; subcategory groups contributing <1.0% not reported; data are Day 1 intakes).

**Table S3.** Subcategory food sources of vitamin D (D2+D3) contribution in the total US diet, adults ≥51 years old (*N =* 4522; gender combined; daily intake data; NHANES 2011-2014; subcategory groups contributing <1.0% not reported; data are Day 1 intakes).

**Table S4.** Subcategory food sources of potassium contribution in the total US diet, adults ≥51 years old (*N =* 4522; gender combined; daily intake data; NHANES 2011-2014; subcategory groups contributing <1.0% not reported; data are Day 1 intakes).

**Table S5.** Subcategory food sources of folate, DFE, contribution in the total US diet, adults ≥51 years old (*N =* 4522; gender combined; daily intake data; NHANES 2011-2014; subcategory groups contributing <1.0% not reported; data are Day 1 intakes).

**Table S6.** Subcategory food sources of iron contribution in the total US diet, adults ≥51 years old (*N =* 4522; gender combined; daily intake data; NHANES 2011-2014; subcategory groups contributing <1.0% not reported; data are Day 1 intakes).

**Table S7.** Subcategory food sources of magnesium contribution in the total US diet, adults ≥51 years old (*N =* 4522; gender combined; daily intake data; NHANES 2011-2014; subcategory groups contributing <1.0% not reported; data are Day 1 intakes).

**Table S8.** Subcategory food sources of vitamin A, RAE, contribution in the total US diet, adults ≥51 years old (*N =* 4522; gender combined; daily intake data; NHANES 2011-2014; subcategory groups contributing <1.0% not reported; data are Day 1 intakes).

**Table S9.** Subcategory food sources of vitamin E, as alpha tocopherol, contribution in the total US diet, adults ≥51 years old (*N =* 4522; gender combined; daily intake data; NHANES 2011-2014; subcategory groups contributing <1.0% not reported; data are Day 1 intakes).

**Table S10.** Subcategory food sources of vitamin C contribution in the total US diet, adults ≥51 years old (*N =* 4522; gender combined; daily intake data; NHANES 2011-2014; subcategory groups contributing <1.0% not reported; data are Day 1 intakes).

**Table S11.** Food sources of total fat contribution in the total US diet, adults ≥51 years old (*N =* 4522; gender combined; daily intake data; NHANES 2011-2014; subcategory groups contributing <1.0% not reported; data are Day 1 intakes).

**Table S12.** Food sources of saturated fat contribution in the total US diet, adults ≥51 years old (*N =* 4522; gender combined; daily intake data; NHANES 2011-2014; subcategory groups contributing <1.0% not reported; data are Day 1 intakes).

**Table S13.** Food sources of added sugar contribution in the total US diet, adults ≥51 years old (*N =* 4522; gender combined; daily intake data; NHANES 2011-2014; subcategory groups contributing <1.0% not reported; data are Day 1 intakes).

**Table S14.** Food sources of sodium contribution in the total US diet, adults ≥51 years old (*N =* 4522; gender combined; daily intake data; NHANES 2011-2014; subcategory groups contributing <1.0% not reported; data are Day 1 intakes).

**Table S15.** Food sources of thiamin contribution in the total US diet, adults ≥51 years old (*N =* 4522; gender combined; daily intake data; NHANES 2011-2014; subcategory groups contributing <1.0% not reported; data are Day 1 intakes).

**Table S16.** Food sources of niacin contribution in the total US diet, adults ≥51 years old (*N =* 4522; gender combined; daily intake data; NHANES 2011-2014; subcategory groups contributing <1.0% not reported; data are Day 1 intakes).

**Table S17.** Food sources of riboflavin contribution in the total US diet, adults ≥51 years old (*N =* 4522; gender combined; daily intake data; NHANES 2011-2014; subcategory groups contributing <1.0% not reported; data are Day 1 intakes).

**Table S18.** Food sources of vitamin B6 contribution in the total US diet, adults ≥51 years old (*N =* 4522; gender combined; daily intake data; NHANES 2011-2014; subcategory groups contributing <1.0% not reported; data are Day 1 intakes).

**Table S19.** Food sources of vitamin B12 contribution in the total US diet, adults ≥51 years old (*N =* 4522; gender combined; daily intake data; NHANES 2011-2014; subcategory groups contributing <1.0% not reported; data are Day 1 intakes).

**Table S1.** Subcategory food sources of dietary fiber contribution in the total US diet, adults ≥51 years old (*N =* 4522; gender combined; daily intake data; NHANES 2011-2014; subcategory groups contributing <1.0% not reported; data are Day 1 intakes).

**Table S2.** Subcategory food sources of calcium contribution in the total US diet, adults ≥51 years old (*N =* 4522; gender combined; daily intake data; NHANES 2011-2014; subcategory groups contributing <1.0% not reported; data are Day 1 intakes).

**Table S3.** Subcategory food sources of vitamin D (D2+D3) contribution in the total US diet, adults ≥51 years old (*N =* 4522; gender combined; daily intake data; NHANES 2011-2014; subcategory groups contributing <1.0% not reported; data are Day 1 intakes).

**Table S4.** Subcategory food sources of potassium contribution in the total US diet, adults ≥51 years old (*N =* 4522; gender combined; daily intake data; NHANES 2011-2014; subcategory groups contributing <1.0% not reported; data are Day 1 intakes).

**Table S5.** Subcategory food sources of folate, DFE, contribution in the total US diet, adults ≥51 years old (*N =* 4522; gender combined; daily intake data; NHANES 2011-2014; subcategory groups contributing <1.0% not reported; data are Day 1 intakes).

**Table S6.** Subcategory food sources of iron contribution in the total US diet, adults ≥51 years old (*N =* 4522; gender combined; daily intake data; NHANES 2011-2014; subcategory groups contributing <1.0% not reported; data are Day 1 intakes).

**Table S7.** Subcategory food sources of magnesium contribution in the total US diet, adults ≥51 years old (*N =* 4522; gender combined; daily intake data; NHANES 2011-2014; subcategory groups contributing <1.0% not reported; data are Day 1 intakes).

**Table S8.** Subcategory food sources of vitamin A, RAE, contribution in the total US diet, adults ≥51 years old (*N =* 4522; gender combined; daily intake data; NHANES 2011-2014; subcategory groups contributing <1.0% not reported; data are Day 1 intakes).

**Table S9.** Subcategory food sources of vitamin E, as alpha tocopherol, contribution in the total US diet, adults ≥51 years old (*N =* 4522; gender combined; daily intake data; NHANES 2011-2014; subcategory groups contributing <1.0% not reported; data are Day 1 intakes).

**Table S10.** Subcategory food sources of vitamin C contribution in the total US diet, adults ≥51 years old (*N =* 4522; gender combined; daily intake data; NHANES 2011-2014; subcategory groups contributing <1.0% not reported; data are Day 1 intakes).

**Table S11.** Food sources of total fat contribution in the total US diet, adults ≥51 years old (*N =* 4522; gender combined; daily intake data; NHANES 2011-2014; subcategory groups contributing <1.0% not reported; data are Day 1 intakes).

**Table S12.** Food sources of saturated fat contribution in the total US diet, adults ≥51 years old (*N =* 4522; gender combined; daily intake data; NHANES 2011-2014; subcategory groups contributing <1.0% not reported; data are Day 1 intakes).

**Table S13.** Food sources of added sugar contribution in the total US diet, adults ≥51 years old (*N =* 4522; gender combined; daily intake data; NHANES 2011-2014; subcategory groups contributing <1.0% not reported; data are Day 1 intakes).

**Table S14.** Food sources of sodium contribution in the total US diet, adults ≥51 years old (*N =* 4522; gender combined; daily intake data; NHANES 2011-2014; subcategory groups contributing <1.0% not reported; data are Day 1 intakes).

**Table S15.** Food sources of thiamin contribution in the total US diet, adults ≥51 years old (*N =* 4522; gender combined; daily intake data; NHANES 2011-2014; subcategory groups contributing <1.0% not reported; data are Day 1 intakes).

**Table S16.** Food sources of niacin contribution in the total US diet, adults ≥51 years old (*N =* 4522; gender combined; daily intake data; NHANES 2011-2014; subcategory groups contributing <1.0% not reported; data are Day 1 intakes).

**Table S17.** Food sources of riboflavin contribution in the total US diet, adults ≥51 years old (*N =* 4522; gender combined; daily intake data; NHANES 2011-2014; subcategory groups contributing <1.0% not reported; data are Day 1 intakes).

**Table S18.** Food sources of vitamin B6 contribution in the total US diet, adults ≥51 years old (*N =* 4522; gender combined; daily intake data; NHANES 2011-2014; subcategory groups contributing <1.0% not reported; data are Day 1 intakes).

**Table S19.** Food sources of vitamin B12 contribution in the total US diet, adults ≥51 years old (*N =* 4522; gender combined; daily intake data; NHANES 2011-2014; subcategory groups contributing <1.0% not reported; data are Day 1 intakes).
